# Effects of different platform preparation and retrieval techniques on broken file removal and fracture strength

**DOI:** 10.1590/1807-3107bor-2026.vol40.047

**Published:** 2026-07-27

**Authors:** Esra YAVAS, Selen KUCUKKAYA EREN

**Affiliations:** (a) Hacettepe University, Faculty of Dentistry, Department of Endodontics, Ankara, Turkey.

**Keywords:** Dentin, Device Removal, Fracture Strength, Root Canal Preparation, Ultrasonics

## Abstract

The aim was to evaluate the effects of different strategies on broken instrument removal and root fracture strength. After canal preparation of anterior single-rooted teeth, the samples were divided into one control group (n = 10) and six experimental groups (n = 15). A #25/.06 rotary file was broken at a length of 4 mm in the middle-third canal region in the experimental groups, and no instrument fractures were created in the control group. Access to the instrument was achieved with Gates-Glidden (GG) burs or ultrasonic tips. Dentin around the instrument was removed to create partial or full platforms. Finally, the instruments were removed with ultrasonic tips or a BTR Pen. The effectiveness of broken instrument removal, the duration of removal, and any complications that occurred were recorded. All the root canals were obturated. Fracture strength analysis was performed using a universal testing machine. Statistical analysis was performed using the Kruskal-Wallis and Dunn tests (p < 0.05). All broken instruments were successfully removed. The access time to the broken instrument in the groups using GG burs was shorter than that in the groups using ultrasonic tips (p < 0.05), while there was no significant difference between the groups in terms of total removal time (p > 0.05). The fracture strength of the control group was similar to that of the group in which ultrasonic tips were used to access the broken instrument and to create a full platform, and the BTR Pen was used for the removal of the broken instrument (p > 0.05), but showed higher fracture strength than that of the other experimental groups (p < 0.05). Different strategies used in the removal of broken instruments affected access time and root fracture strength.

## Introduction

Thorough cleaning and shaping of the root canal system is an essential step in endodontic treatment.^1^ Blockages caused by broken instruments during shaping may hinder the complete removal of infected tissues and microorganisms from the canal system.^2^ The ideal management of such situations involves removing the broken instrument from the canal, followed by thorough cleaning, shaping, and filling of the root canal.^3^ However, complications may occur during the removal of broken instruments, such as excessive removal of dentin, ledge formation, perforation, and pushing the broken fragment to the periapical area.^4^

To remove a broken instrument from the root canal, a staging platform is initially created using Gates Glidden (GG) burs or ultrasonic tips to provide straight access to the fragment.^5^ A staging platform can be prepared by completely or partially removing dentin around the broken instrument.^6^ A full platform is created by removing dentin to completely expose the coronal portion of the fractured instrument, while a partial platform is prepared to expose the coronal portion of the fractured instrument on the inner wall by 180°.^6^ In a recent case report, preparing a partial platform was recommended to avoid ledge formation, transportation, and perforation.^6^

Following the creation of a staging platform, activated ultrasonic tips are placed between the fragment and dentin to loosen the broken instrument. In some cases, the broken fragment cannot be removed from the root canal even if it becomes loose, and excessive ultrasonic use may result in iatrogenic errors. In such instances, the use of other instrument removal techniques can be considered.^5^ The Broken Tool Remover Pen (BTR Pen) (Cerkamed Medical Company, Stalowa Wola, Poland) is a system that can facilitate the removal procedure by grasping the broken instrument with its nitinol loop.^7^ This system includes three tips with diameters of 0.3, 0.4, and 0.5 mm and a needle. Once the broken instrument is coronally exposed, the lasso is formed by the system needle and placed around the fragment to provide a solid grip.^7^

Techniques that prevent excessive dentin removal during broken instrument removal may facilitate the procedure and prevent complications, thus reducing the risk of root fracture. According to a recent systematic review, broken instrument removal often results in significant dentin removal, especially when fragments are located in the middle or apical thirds of the root, which is associated with reduced fracture resistance.^8^ Ultrasonic removal of broken files from the middle third of root canals has been shown to reduce the root fracture resistance, primarily because of increased dentin loss, compared with teeth in which no instrument separation occurred.^9^ In another study, ultrasonic removal resulted in more dentin thinning than combined ultrasonic/lasso techniques, suggesting that hybrid approaches may better preserve root structure.^10^ Furthermore, a previous micro-CT study revealed that broken instrument removal with ultrasonic tips can create new dentinal microcracks, potentially compromising long-term structural integrity.^11^ Nevertheless, ultrasonic removal of broken files was associated with significantly higher fracture resistance than the use of instrument removal kits^12^ or static endodontic guides.^13^ The choice of technique should be carefully balanced to achieve successful removal while preserving as much dentin as possible to reduce the risk of root fracture. The aim of this study was to evaluate the effects of preparing partial and full staging platforms with different instruments and using ultrasonic tips and a BTR Pen on broken instrument removal and root fracture strength. The null hypothesis was that different strategies for broken instrument removal do not affect removal success, operation time, or root fracture strength.

## Methods

The study protocol was approved by the Ethics Committee of Hacettepe University (number: GO 21/1360). The sample size was determined based on the results of previous studies using G*Power 3.1 software (Heinrich Heine University, Düsseldorf, Germany).^9,14^ Analysis with 90% power and 5% type I error revealed that at least 11 samples per group were required for the determination of the efficacy and duration of the removal procedure, and 9 samples were required for the fracture strength test. Accordingly, 15 samples in each experimental group were considered sufficient in terms of statistical power.

### Selection and preparation of samples

One hundred freshly extracted human anterior single-rooted teeth with similar root lengths and diameters were used. Periapical radiographs were taken from the mesiodistal and buccolingual directions to confirm the presence of a single canal. The selected teeth had mature roots and no root defects, including caries, resorption, fractures or cracks.

The tooth length was standardized to 15 mm by removing the coronal tooth structure. The apical patency of each canal was checked with a #15 K-type file (Dentsply Sirona, Ballaigues, Switzerland). Working length (WL) was established at 1 mm short of the apical foramen using a #15 K-type file (Dentsply Sirona). Each sample was placed in acrylic resin (Meliodent, Bayer Co., Leverkusen, Germany) with the coronal surface exposed. The coronal third of each root canal was shaped with the Sx rotary file of the ProTaper Universal file system (Dentsply Sirona) attached to an endodontic motor (X-Smart Plus, Dentsply Sirona, Ballaigues, Switzerland). Afterward, the root canals were instrumented using #15 and #20 K-type files (Dentsply Sirona) and irrigated with 2 ml of 5.25% sodium hypochlorite (NaOCl) after each file. At this stage, 10 teeth were randomly allocated to the control group, and no instrument fractures were created.

A #25/.06 rotary file (Endoart, İnci Dental, Istanbul, Turkey) was notched to half the instrument thickness with a diamond disc at 4 mm from the tip. Afterward, the instrument attached to an endodontic motor was rotated clockwise with pressure so that it separated in the middle third of the root canal. A periapical radiograph was taken from each sample to confirm the position of the separated instrument.

The remaining 90 teeth were randomly divided into 6 experimental groups (n = 15) as follows:

- Group GFU: Coronal access to the broken fragment was made with modified GG burs (no 2–4) (Dentsply Sirona). A full platform was prepared with an ET40 ultrasonic tip (Satelec, Merignac, France) to circumferentially expose the coronal third of the fractured instrument. An ET25 ultrasonic tip (Satelec) was used to remove the broken fragment.
- Group GFB: Coronal access to the broken fragment was made with modified GG burs (no 2–4) (Dentsply Sirona). A full platform was prepared with an ET40 ultrasonic tip (Satelec) to circumferentially expose the coronal third of the fractured instrument. BTR Pen (Cerkamed Medical Company) was used to remove the broken fragment.
- Group GPU: Coronal access to the broken fragment was made with modified GG burs (no 2–4) (Dentsply Sirona). A partial platform was prepared with an ET40 ultrasonic tip (Satelec) to expose the coronal third of the fractured instrument on the inner wall by 180°. An ET25 ultrasonic tip (Satelec) was used to remove the broken fragment.
- Group UFU: Coronal access to the broken fragment was achieved with an ET20 ultrasonic tip (Satelec) connected to the piezoelectric unit. A full platform was prepared with an ET40 ultrasonic tip (Satelec) to circumferentially expose the coronal third of the fractured instrument. An ET25 ultrasonic tip (Satelec) was used to remove the broken fragment.
- Group UFB: Coronal access to the broken fragment was achieved with an ET20 ultrasonic tip (Satelec) connected to the piezoelectric unit. A full platform was prepared with an ET40 ultrasonic tip (Satelec) to circumferentially expose the coronal third of the fractured instrument. BTR Pen was used to remove the broken fragment.
- Group UPU: Coronal access to the broken fragment was achieved with an ET20 ultrasonic tip (Satelec) connected to the piezoelectric unit. A partial platform was prepared with an ET40 ultrasonic tip (Satelec) to expose the coronal third of the fractured instrument on the inner wall by 180°. An ET25 ultrasonic tip (Satelec) was used to remove the broken fragment.

**Figure 1:**
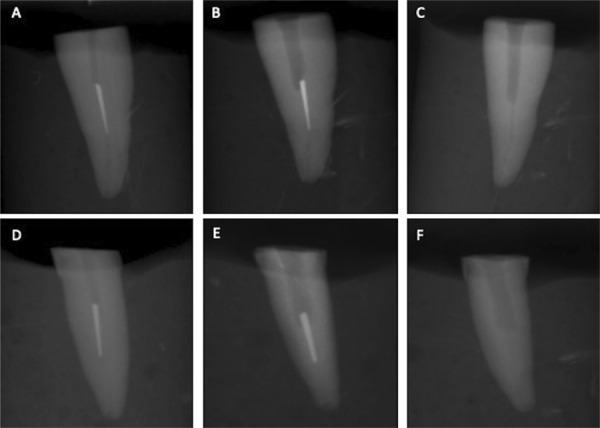
Representative periapical radiographs of the experimental procedures. After an instrument fracture was created (A), access to the instrument was obtained, a partial platform was prepared (B), and the instrument was removed with ultrasonic tips (C). After an instrument fracture was created (D), access to the instrument was obtained, a full platform was prepared (E), and the instrument was removed with a BTR Pen (F).

The GG burs were modified by cutting them perpendicular to the long axis at the largest cross-sectional diameter. The ultrasonic tips were used with a piezoelectric unit (Newtron Booster, Satelec) at a low power setting. To prevent temperature increase, each ultrasonic activation was limited to 15 seconds, and the root canals were rinsed with 2 ml of 5.25% NaOCl during these intervals. The instrument removal procedures were performed by a single operator under magnification using a dental operating microscope (ZEISS OPMI PROergo, Zeiss Group, Oberkochen, Germany). Representative periapical radiographs of the experimental procedures are shown in Figure 1.

**Figure 2:**
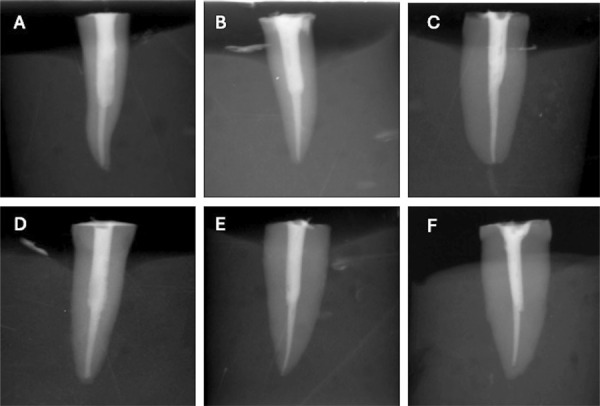
Periapical radiographs of the teeth with root canal filling after removal of the broken instrument. A: GFU, B: GFB, C: GPU, D: UFU, E: UFB, F: UPU.

After broken instrument removal, each root canal, including the control group, was shaped sequentially with rotary instruments of sizes 10/.06, 15/.06, 20/.06, 25/.06, and 30/.06 (Endoart). The root canals were irrigated with 2 ml of 5.25% NaOCl after each instrument. Final irrigation was performed with 5 ml of 5.25% NaOCl, 5 ml of 17% EDTA, and 5 ml of distilled water. Afterward, each root canal was dried with paper points (Dentsply Sirona) and obturated using a #30/.06 gutta-percha (Endoart) and an epoxy resin-based root canal sealer (AH Plus, Dentsply Sirona). A periapical radiograph was taken from each sample to confirm the quality of obturation (Figure 2). All the samples were kept in a moist environment throughout the experiments.

### Time

The maximum time to remove the broken instrument from the root canal was determined as 45 minutes. If the instrument could not be removed from the root canal within this time, it was considered a failure. The removal of the broken instrument from the root canal (success/failure), the time to access the broken instrument and the time to remove it from the root canal were recorded in an Excel file. The total time was calculated as the sum of these two time periods. In addition, complications such as ledge formation, root perforation, or secondary fractures of the broken instrument were also recorded if they occurred.

### Fracture strength analysis

A Universal Testing Machine (LLYOD LRX, Worthing, West Sussex, England) was used to apply a vertical loading. A 4-mm diameter circular tip was centered over the canal orifice, and a slowly increasing vertical force parallel to the long axis of the tooth was exerted (1 mm/min) until fracture occurred. When the fracture occurred, the force was recorded in Newtons. The fracture directions were also recorded for each sample. One operator who was blinded to the groups assessed the outcome.

### Statistical analysis

The data were analyzed using the Kruskal-Wallis and Dunn tests. The level of significance was set at p < 0.05. The analyses were performed using a software program (IBM SPSS Statistics 23.0, SPSS Inc*.*, Chicago, USA).

## Results

Table 1 presents the findings regarding the removal of fractured instruments from the root canal for each group. All the fractured instruments were successfully removed from the canals in each group. There was a significant difference in the time required to access the broken instrument between the groups (p < 0.05), while there was no significant difference between the groups in terms of instrument retrieval time or total time (p > 0.05). Pairwise comparisons revealed that the time required to access the broken instrument was significantly shorter for the GFU and GPU groups than for the UFU and UFB groups (p < 0.05), while there was no significant difference among the other groups (p > 0.05).

**Table 1:** Results regarding the removal of broken instruments from the root canal

Groups	Success rate (%)	Time (mean ± standard deviation)		
		Access time	Instrument removal time	Total time
GFU	100%	4.55^a^± 1.55	14.58^a^ ± 9.14	19.13^a^ ± 9.81
GFB	100%	4.80^a,b^± 1.49	12.93^a^ ± 7.09	17.73^a^ ± 7.14
GPU	100%	4.52^a^± 1.28	16.20^a^± 7.99	20.72^a^ ± 8.69
UFU	100%	6.31^b^± 1.26	11.80^a^ ± 5.30	18.11^a^ ± 5.52
UFB	100%	6.52^b^± 1.81	12.09^a^ ± 3.95	18.61^a^ ± 4.30
UPU	100%	5.80^a,b,^± 1.87	11.79^a^ ± 6.03	17.59^a^ ± 6.71

Different letters within each column indicate significant differences between groups (p < 0.05).

During the removal procedures, fractured instruments were pushed into the apical third region of the root canal in 9 samples: 2 in the GFU group, 3 in the GFB group, and 4 in the GPU group. Thereafter, these instruments were successfully removed from the canals. In 2 samples in the GPU group, secondary fractures occurred in the broken fragments during the application of ultrasonic energy, and subsequently, these fragments were also successfully removed from the canals. In 1 sample each in the GFU, GFB, and UPU groups, a ledge occurred in the canal during instrument removal.

**Figure 3:**
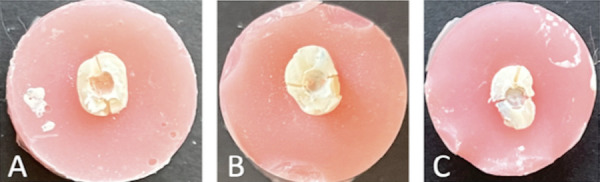
Photographs showing root fractures in the buccolingual (A), mesiodistal (B) and both (C) directions after fracture strength analysis.

Table 2 presents the results of fracture resistance analysis. No significant difference was found between the control group and the UFB group (p > 0.05), while the control group exhibited significantly higher fracture resistance compared to the other experimental groups (p < 0.05). The UFB group demonstrated significantly higher fracture resistance than the GFU group (p < 0.05), while there were no significant differences between the other experimental groups (p > 0.05). A total of 50 root fractures were in the buccolingual direction, 11 root fractures were in the mesiodistal direction and 39 root fractures were in both directions (Figure 3).

**Table 2:** Results of fracture strength analysis.

Groups	Fracture Strenght (N)	Fracture Directions (number of samples)		
	(mean±standard deviation)	BL	MD	Combined (BL + MD)
Control	535.34^a^ ± 69.56	5	1	4
GFU	220.21^b^ ± 108.56	10	-	5
GFB	266.17^b,c^ ± 115.93	7	2	6
GPU	271.58^b,c^ ± 117.61	8	1	6
UFU	352.35^b,c^ ± 128.62	6	2	7
UFB	384.32^a,c^ ±162.32	7	2	6
UPU	345.47^b,c^ ± 145.18	7	3	5

BL: Buccolingual;MD: Mesiodistal. Different letters within each column indicate significant differences between groups (p < 0.05). There is no statistically significant difference between the groups sharing the same letter (p > 0.05).

## Discussion

In this study, different strategies, including preparing different types of platforms and using different instruments for the removal of broken instruments from the root canal, were evaluated in terms of removal success, operation time, and root fracture strength. Although all the broken instruments were successfully removed, differences in the time required to access the broken fragment and in root fracture strength were detected between the groups. Therefore, the null hypothesis was partially rejected.

Removal of broken instruments, especially from the middle and apical thirds of the root canal, has been reported to reduce root fracture strength.^4,9^ Similarly, the control group presented the highest fracture strength values in the current study. On the other hand, despite the lower fracture strength values in the UFB group, there was no significant difference between the UFB group and the control group. This could be related to minimal dentin removal around the broken instrument due to the use of the BTR Pen in the UFB group. Exposing one-third of the broken instrument is technically sufficient for loop devices such as the BTR Pen to grasp and remove the fractured fragment from the canal.^5^ Similar to the present findings, a previous study reported that the use of a loop device in the removal of a fractured instrument from the middle third of the root canal helps preserve dentin.^15^ When only ultrasonic tips are used during the removal procedure, more dentin removal toward the apex may be necessary to loosen the fractured instrument. This may explain the lower fracture strength values in the groups where only ultrasonic tips were used to remove broken instruments. Notably, compared with the other groups, the groups using GG burs tended to have lower fracture strength values. This can be attributed to the higher incidence of crack formation and dentin defects associated with GG burs.^16,17^ It has been reported that creating a partial platform at the coronal end of a fractured instrument can help preserve the remaining dentin structure during its retrieval from the canal.^6^ Although the concept of the full platform requires more dentin removal circumferentially than the partial platform, platform type had no significant effect on root fracture strength in the present study. This may be due to the removal of additional dentin toward the apex in the partial platform groups to mobilize the fractured fragment. Notably, the BTR Pen was not used in the partial platform groups, as complete circumferential dentin removal is needed to effectively grasp the coronal portion of the fractured instrument.

In studies evaluating root fracture strength, all controllable factors should be standardized.^18^ In the present study, single-rooted teeth with a single canal that had similar widths and lengths were used to minimize anatomical variations. The removal of fractured instruments from the apical region of the root canal is difficult and has a high risk of complications such as perforation, ledge formation, and excessive dentin loss.^4,19,20^ Therefore, if the instrument is broken in the apical root canal region during the final stage of root canal instrumentation, it is recommended to obturate the root canal up to the fractured fragment or bypass it rather than to remove it.^21-23^ On the other hand, instruments that are broken in the middle third of the root canal should be removed so that the cleaning, shaping, and filling steps can be completed properly.^9^ Instruments with a larger cross-sectional area are more prone to breakage in the root canal because of lower cyclic fatigue resistance and greater stress.^24, 25^ Therefore, for clinical significance, a #25/.06 rotary file was broken in the middle third of each root canal in this study. Attempts to remove fractured instruments from root canals should not take longer than 45–60 minutes, as success rates may decrease with increased time because of the risk of complications.^19^ Therefore, in this study, a maximum removal time of 45 minutes was used, and all fractured instruments were successfully removed within this time period.

Previous studies reported success rates between 88% and 95% in removing broken instruments using ultrasonic tips alone,^26-28^ whereas the use of a loop device with ultrasonic tips achieved a 100% success rate.^29^ In the present study, no difference was found in effectiveness between the use of ultrasonic tips alone and the use of the BTR Pen with ultrasonic tips, as all broken instruments were successfully removed. Based on the present findings, the time to access the broken fragment with GG burs was shorter than that with ultrasonic tips. This may be due to faster dentin removal by the rotary motion of GG burs and the need for cooling during ultrasonic energy application. However, no significant difference was observed between the groups in terms of the time to remove the broken instrument and the total time, similar to the findings of previous studies.^15,30^ In the literature, the total time to remove broken instruments from the middle third of the root canal varies from 15 to 42 minutes.^15,30,31^ In this study, the broken instruments were removed from the middle third of the root canals in an average of 18.65 minutes. Variations in time among studies may be due to factors such as the curvature and diameter of the root canal, the type and length of the broken instrument, the technique used to remove the broken instrument, and operator experience.

Various complications can occur during the removal of a broken instrument from the root canal, the most common of which is excessive dentin loss.^32^ This loss may lead to decreased root fracture strength^33^ and increased susceptibility to vertical root fractures over time.^4,34^ Vertical root fractures frequently occur in the buccolingual direction.^31^ Similarly, in the present study, fractures were observed in the buccolingual direction in half of the samples.

During the removal procedure, the broken instruments were pushed into the apical third region in some samples. Interestingly, all these samples were in the groups prepared with GG burs. This may be related to the rotational movement and operating speed of GG burs, and their larger diameter compared to the broken instrument. In the present study, secondary fracture of the instrument occurred in 2 samples. The reasons for this may include structural defects occurring in NiTi instruments during the production phase, direct contact of the ultrasonic tip with the broken instrument, and changes in the NiTi alloy due to increased temperature after ultrasonic application. To prevent secondary fractures, ultrasonic tips were used at low power settings, ultrasonic energy was applied intermittently, and frequent irrigation was performed. In the present study, secondary fractures were rarely seen. Interestingly, all of them were seen in the GPU group, which may be related to the application of the ultrasonic tip from the same side of the fractured instrument throughout the procedure due to the partial platform and the application of ultrasonic energy for a longer period compared to the groups using the BTR Pen. Moreover, no root perforation occurred during the removal of broken instruments from the root canal. Performing the procedures under magnification using a dental operating microscope and using ultrasonic tips at low power settings may have contributed to this outcome.

Instrument fractures frequently occur in multirooted teeth with curvatures.^20, 35^ In a recent study, the incidence of instrument fractures in incisors and premolars was reported to be between 0.9% and 2.2%, while in molars it was reported to be 6.9%.^35^ In the present study, the use of single- and straight-rooted teeth could be considered a limitation in terms of representing a less common situation. These teeth were used in this laboratory study to allow for a standardized comparison of different techniques without being affected by anatomical difficulties. Further studies may evaluate the effectiveness of these techniques in more complex cases.

## Conclusion

On the basis of the findings of the present study, accessing the broken instrument with ultrasonic tips, creating a full platform and removing the broken instrument from the canal using BTR Pen can be recommended for clinical applications.

## Data Availability

The authors declare that all data generated or analyzed during this study are included in this published article.

## References

[1] Paqué F, Balmer M, Attin T, Peters OA (2010). Preparation of oval-shaped root canals in mandibular molars using nickel-titanium rotary instruments: a micro-computed tomography study. J Endod.

[2] Lin LM, Rosenberg PA, Lin J (2005). Do procedural errors cause endodontic treatment failure?. J Am Dent Assoc.

[3] Madarati AA, Hunter MJ, Dummer PM (2013). Management of intracanal separated instruments. J Endod.

[4] Souter NJ, Messer HH (2005). Complications associated with fractured file removal using an ultrasonic technique. J Endod.

[5] Ruddle CJ (2004). Nonsurgical retreatment. J Endod.

[6] Narasimhan B, Vinothkumar TS, Praveen R, Setzer FC, Nagendrababu V (2021). A modified partial platform technique to retrieve instrument fragments from curved and narrow canals: a report of 2 cases. J Endod.

[7] BTR PEN (2021). Broken Tool Removal System. Stalowa Wola.

[8] Portela NN, Rech JP, Marchionatti AM, Barasuol JC (2022). Techniques to address fractured instruments in the middle or apical third of the root canal in human permanent teeth: a systematic review of the in vitro studies. Clin Oral Investig.

[9] Fu M, Huang X, Zhang K, Hou B (2019). Effects of ultrasonic removal of fractured files from the middle third of root canals on the resistance to vertical root fracture. J Endod.

[10] Versiani MA, Dias HS, Silva EJ, Belladonna FG, Martins JN, De-Deus G (2025). The cost of instrument retrieval on the root integrity. Int Endod J.

[11] Fu M, Huang X, He W, Hou B (2018). Effects of ultrasonic removal of fractured files from the middle third of root canals on dentinal cracks: a micro-computed tomography study. Int Endod J.

[12] Gencoglu N, Sivet C (2018). Fracture resistance of roots obturated by different techniques after the removal of broken instruments. Eur J Res Dent.

[13] Yassa SA, Nabeel M, Ghobashy AM, Alkhawas MB (2025). Fracture resistance and volumetric dentin change after management of broken instrument using static navigation: an in vitro study. J Conserv Dent Endod.

[14] Yang Q, Shen Y, Huang D, Zhou X, Gao Y, Haapasalo M (2017). Evaluation of two trephine techniques for removal of fractured rotary nickel-titanium instruments from root canals. J Endod.

[15] Abdeen MA, Plotino G, Hassanien EE, Turky M (2023). Evaluation of dentine structure loss after separated file retrieval by three different techniques: an ex-vivo study. Eur Endod J.

[16] Arslan H, Karatas E, Capar ID, Ozsu D, Doganay E (2014). Effect of ProTaper Universal, Endoflare, Revo-S, HyFlex coronal flaring instruments, and Gates Glidden drills on crack formation. J Endod.

[17] Shemesh H, Bier CA, Wu MK, Tanomaru-Filho M, Wesselink PR (2009). The effects of canal preparation and filling on the incidence of dentinal defects. Int Endod J.

[18] Uzunoglu-Özyürek E, Küçükkaya Eren S, Karahan S (2018). Effect of root canal sealers on the fracture resistance of endodontically treated teeth: a systematic review of in vitro studies. Clin Oral Investig.

[19] Suter B, Lussi A, Sequeira P (2005). Probability of removing fractured instruments from root canals. Int Endod J.

[20] Nevares G, Cunha RS, Zuolo ML, Bueno CE (2012). Success rates for removing or bypassing fractured instruments: a prospective clinical study. J Endod.

[21] Simon S, Machtou P, Tomson P, Adams N, Lumley P (2008). Influence of fractured instruments on the success rate of endodontic treatment. Dent Update.

[22] Parashos P, Messer HH (2006). Rotary NiTi instrument fracture and its consequences. J Endod.

[23] Ward JR, Parashos P, Messer HH (2003). Evaluation of an ultrasonic technique to remove fractured rotary nickel-titanium endodontic instruments from root canals: clinical cases. J Endod.

[24] Ullmann CJ, Peters OA (2005). Effect of cyclic fatigue on static fracture loads in ProTaper nickel-titanium rotary instruments. J Endod.

[25] Pedullà E, La Rosa GR, Virgillito C, Rapisarda E, Kim HC, Generali L (2020). Cyclic fatigue resistance of nickel-titanium rotary instruments according to the angle of file access and radius of root canal. J Endod.

[26] Fu M, Zhang Z, Hou B (2011). Removal of broken files from root canals by using ultrasonic techniques combined with dental microscope: a retrospective analysis of treatment outcome. J Endod.

[27] Pruthi PJ, Nawal RR, Talwar S, Verma M (2020). Comparative evaluation of the effectiveness of ultrasonic tips versus the Terauchi file retrieval kit for the removal of separated endodontic instruments. Restor Dent Endod.

[28] Cujé J, Bargholz C, Hülsmann M (2010). The outcome of retained instrument removal in a specialist practice. Int Endod J.

[29] Terauchi Y, Sexton C, Bakland LK, Bogen G (2021). Factors affecting the removal time of separated instruments. J Endod.

[30] Dulundu M, Helvacioglu-Yigit D (2022). The Efficiency of the BTR-Pen System in removing different types of broken instruments from root canals and its effect on the fracture resistance of roots. Materials (Basel).

[31] Madarati AA, Qualtrough AJ, Watts DC (2010). Vertical fracture resistance of roots after ultrasonic removal of fractured instruments. Int Endod J.

[32] Madarati AA, Watts DC, Qualtrough AJ (2008). Opinions and attitudes of endodontists and general dental practitioners in the UK towards the intra-canal fracture of endodontic instruments. Part 2. Int Endod J.

[33] Garg H, Grewal MS (2016). Cone-beam computed tomography volumetric analysis and comparison of dentin structure loss after retrieval of separated instrument by using ultrasonic EMS and ProUltra tips. J Endod.

[34] Lertchirakarn V, Palamara JE, Messer HH (2003). Patterns of vertical root fracture: factors affecting stress distribution in the root canal. J Endod.

[35] Eskibaglar M, Özata MY, Ocak MS, Öztekin F (2023). Investigation of fracture prevalence of instruments used in root canal treatments at a faculty of dentistry: a prospective study. Restor Dent Endod.

